# Mapping polarization induced surface band bending on the Rashba semiconductor BiTeI

**DOI:** 10.1038/ncomms5066

**Published:** 2014-06-05

**Authors:** Christopher John Butler, Hung-Hsiang Yang, Jhen-Yong Hong, Shih-Hao Hsu, Raman Sankar, Chun-I Lu, Hsin-Yu Lu, Kui-Hon Ou Yang, Hung-Wei Shiu, Chia-Hao Chen, Chao-Cheng Kaun, Guo-Jiun Shu, Fang-Cheng Chou, Minn-Tsong Lin

**Affiliations:** 1 Department of Physics, National Taiwan University, Taipei 10617, Taiwan; 2 Center for Condensed Matter Sciences, National Taiwan University, Taipei 10617, Taiwan; 3 Institute of Atomic and Molecular Sciences, Academia Sinica, Taipei 10617, Taiwan; 4 National Synchrotron Radiation Research Center, Hsinchu 30076, Taiwan; 5 Research Center for Applied Sciences, Academia Sinica, Taipei 11529, Taiwan; 6 Department of Physics, National Tsing Hua University, Hsinchu 30013, Taiwan; 7 Taiwan Consortium of Emergent Crystalline Materials (TCECM), National Science Council, Taipei 10622, Taiwan

## Abstract

Surfaces of semiconductors with strong spin-orbit coupling are of great interest for use in spintronic devices exploiting the Rashba effect. BiTeI features large Rashba-type spin splitting in both valence and conduction bands. Either can be shifted towards the Fermi level by surface band bending induced by the two possible polar terminations, making Rashba spin-split electron or hole bands electronically accessible. Here we demonstrate the first real-space microscopic identification of each termination with a multi-technique experimental approach. Using spatially resolved tunnelling spectroscopy across the lateral boundary between the two terminations, a previously speculated on p-n junction-like discontinuity in electronic structure at the lateral boundary is confirmed experimentally. These findings realize an important step towards the exploitation of the unique behaviour of the Rashba semiconductor BiTeI for new device concepts in spintronics.

## Abstract (web summary)

Surfaces of semiconductors exhibiting large Rasha effect are of great interest for spintronics applications. Here, Butler *et al*. present the spectroscopic observation and microscopic mapping of termination-dependent band-bending at the surface of Rashba semiconductor BiTeI.

## Graphical abstract

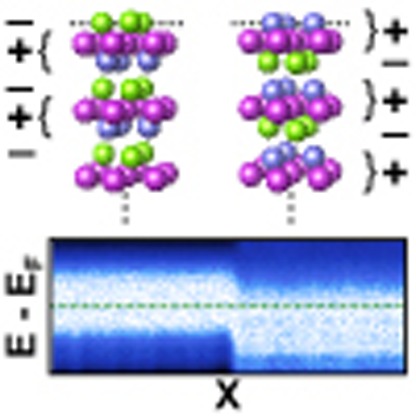

High-Z materials have attracted great interest due to their strong spin-orbit interaction, which is the basis of several proposed spintronic devices featuring electric control of spin currents, a major goal of spintronics^1^,^2^,^3^. Such devices generally exploit the Rashba effect, first described in bulk systems by the Dresselhaus^4^ and Rashba^5^,^6^ models, and at surfaces and interfaces by the Rashba-Bychkov model^7^. The strong spin-orbit coupling of high-Z materials serves to enhance the Rashba spin splitting, which, in proposed devices such as spinFETs, is desirable to decrease the spin precession time to below the spin relaxation time^8^.

A number of surfaces such as that of Bi, or those incorporating high-Z atoms by alloying Bi or Pb with noble metals, have been shown to exhibit a large Rashba effect^9^,^10^,^11^,^12^,^13^. However, such materials are based on metal substrates, which inhibit tuning of the spin splitting using an external electric field, and also carry spin-degenerate bulk currents, limiting their utility for spintronics. Hence, there is a need to investigate semiconductor materials that feature a large Rashba spin splitting, particularly in cases where splitting in both valence and conduction bands can provide versatility in tuning of spin transport properties.

BiTeI is a semiconductor that exhibits a giant Rashba spin splitting at the surface and also in the bulk, due to the lack of spatial inversion symmetry of the lattice^14^. Interestingly, for BiTeI it has been shown not only that both conduction and valence bands exhibit Rashba spin splitting, but also that either band, depending on the surface termination, can be shifted so that it crosses the Fermi level near the surface^15^,^16^. Because of the layered polar nature of the BiTeI structure, the crystal can terminate with a layer of either positive or negative nominal charge. The polarization associated with a surface of positive (or negative) charge subjects near-surface electrons to a lowered (raised) Coulomb energy, inducing surface band bending causing all bands to shift sufficiently that the conduction (valence) band edge resides around the Fermi level. This effect, together with both valence and conduction bands’ Rashba spin splitting, allows a choice of spin-polarized bands at the Fermi level.

BiTeI crystals are composed of a sequence of Bi, Te and I layers, with a structure as shown in Fig. 1a. The Bi and Te bond covalently, forming a bilayer which itself bonds ionically to the I, forming a polar structure of (BiTe)^+^ and I^−^ layers^17^ as illustrated in Fig. 1b. These BiTeI trilayers are themselves bonded by the van der Waals force, with the weakly bonded plane between Te and I providing the cleavage planes of the crystal. On this view, cleavage is expected to reveal either a Te or I termination for a given surface.

Previous work by Crepaldi *et al*.^16^, using angle-resolved photoemission spectroscopy, has revealed the spin-dependent dispersion behaviour associated with such terminations. It was noted that patterning of BiTeI surface terminations would open new perspectives for the manipulation of spin-polarized bands. Real-space nano-scale microscopic studies of the spatial distributions of these terminations and the boundaries between them will provide insights into the functional properties of patterned BiTeI surfaces, which may become available in the future.

In this work, we present a microscopic investigation of the spatial distribution of two different chemical terminations found on the vacuum-cleaved BiTeI surface. Scanning tunnelling microscopy (STM) measurements reveal a chemical contrast between two types of regions found, indicating two different surface terminations. The chemical identities of these terminations are assigned by using a combination of X-ray photoemission spectroscopy (XPS) and comparison of measured scanning tunnelling spectroscopy (STS) with the projected density of states (PDOS), calculated using density functional theory (DFT), at the termination layer. Finally, we observe the nano-scale evolution of electronic structure across the boundary between Te and I terminated surfaces using spatially resolved tunnelling spectroscopy, revealing a clear contrast in the sign of the surface band bending at the boundary between the two terminations.

## Results

### STM and conductance imaging

**Figure 1:**
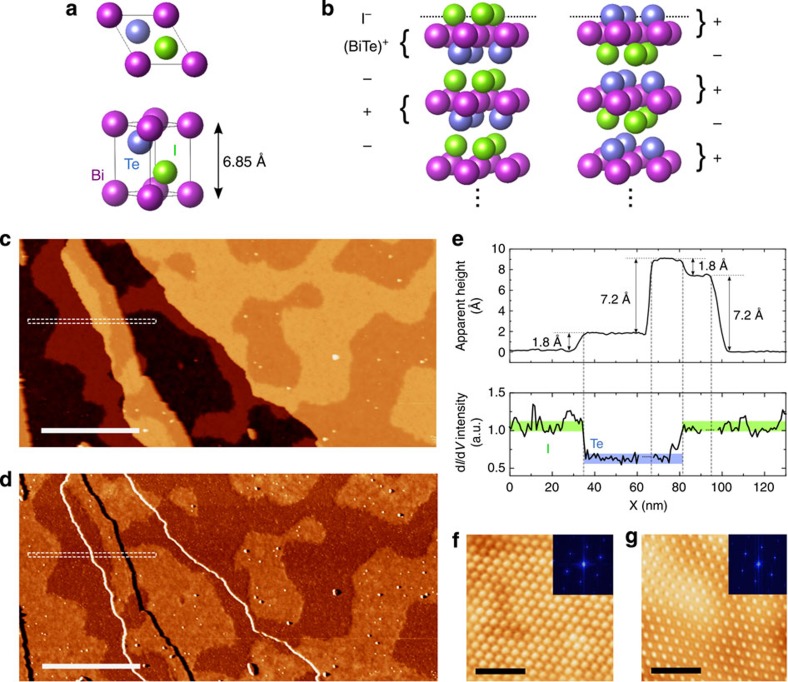
STM observation of two surface terminations on cleaved BiTeI surfaces. (**a**) Schematic representations of the BiTeI primitive cell. (**b**) Illustration of the two expected surface termination structures of BiTeI, with layer-by-layer charges in the ionic limit labelled. (**c**) Large-scale STM topography map (*V*_*bias*_=0.7 V, *I*_*set*_=0.3 nA) for a typical vacuum-cleaved BiTeI surface. Scale bar, 100 nm. (**d**) Simultaneously acquired d*I*/d*V* map. (**e**) Averaged topographic line profile taken along the dashed rectangle in **c**, and averaged d*I*/d*V* intensity taken along the dashed rectangle in **d** (scanning artifacts at step edges have been removed for clarity). The chemical identities assigned to the areas of different conductance are indicated, with identification derived from spectroscopic measurements (see Figs 2 and 3). **f**,**g** show zoom-in STM images and their 2D-FFT images, taken in regions that in conductance maps appear bright and dark, respectively. Scale bar, 2 nm.

STM topography images of the surface, as shown in Fig. 1c, reveal two types of large-scale topographic features characterized by their associated step heights. The larger of the two step types has a height of around 7.20 Å as seen in Fig. 1e. This corresponds closely to the **c** axis lattice parameter *c*=6.85 Å, with the additional height expected due to surface relaxations, and so indicates single unit-cell steps. The lateral distances between these unit-cell steps can exceed 10 μm. The irregularly shaped mesa-like features on the areas between unit-cell steps have a smaller step height accompanied by a clear contrast in differential conductance maps (Fig. 1d) acquired simultaneously with topography maps. This indicates a change in surface termination from one to another of the constituent elements of the crystal. A collection of large-scale conductance maps amounting to a total surface area of 5 × 10^6^ nm^2^ were analysed with suitable image processing software^18^ to determine the ratio of the areas of the two terminations. The percentage of coverage for each termination was consistently seen to converge on a value of 50(±2)% for representative samples of total area 10^5^ nm^2^. Zoom-in images on each termination (Fig. 1f,g) reveal the surface atomic features, displaying the triangular surface lattice and showing that the surface retains good crystallinity on both terminations after cleavage.

No discernible difference could be found between the cleaved surfaces of BiTeI crystals produced by the chemical vapour transport (CVT) and melt growth methods. Furthermore, aside from a somewhat increased roughness for surfaces formed by room temperature cleavage, there was no effect of the cleavage temperature, or of annealing to room temperature after low temperature cleavage, on the resulting surface morphology as described above.

### XPS characterization

**Figure 2:**
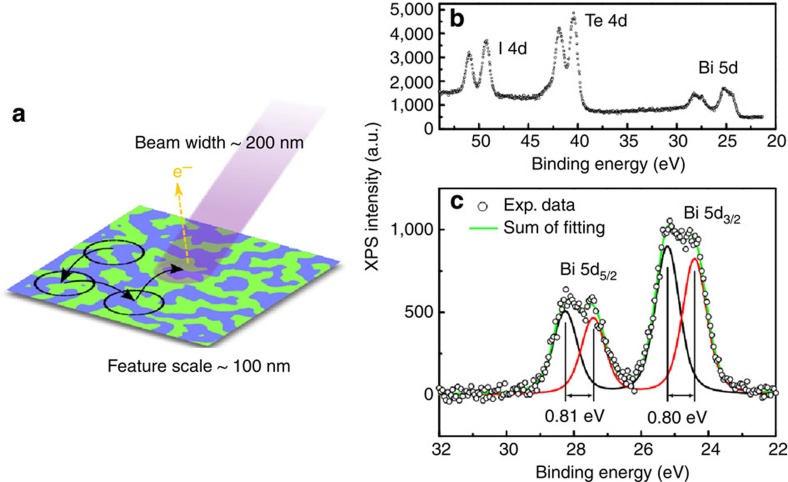
Photoemission spectra for a cleaved BiTeI surface. (**a**) An illustration of the XPS measurement using SPEM. (**b**) XPS spectrum showing peaks for the constituent elements of BiTeI. (**c**) Zoom-in on the Bi 5d peaks, showing the separation into two contributions with a splitting in binding energy of about 0.8 eV.

Though d*I*/d*V* maps indicate a clear chemical contrast between the two regions, STM lacks the element specificity to determine the chemical identity of the terminations. To this end, XPS measurements were performed, with the X-ray beam incident on both terminations, and yielding a total photoemission signal with a roughly even contribution from each (see methods section). XPS spectra, shown in Fig. 2, show relatively suppressed Bi peaks in relation to peaks for Te and I. The Bi peak exhibits a splitting in energy of around 0.8 eV, as seen in Fig. 2b. A similar splitting in the Te 4d and I 4d peaks is also seen in XPS data measured by Crepaldi *et al*.^16^ but not in the data presented in this work. A simple model (see Supplementary Fig. 1) that accounts for differences in experimental details shows that the use of a higher photon energy, resulting in greater sensitivity to sub-surface layers, and a lower spectral resolution account for the loss of these details in the data presented here.

The splitting in the Bi 5d peaks is attributed to the differing surface polarizations of the two terminations represented in the total signal. Photoelectrons from Bi in the Te terminated surface are expected to have a relatively increased binding energy, while those from Bi in the I terminated surface have a lowered binding energy. Because the signals from Bi in the two terminations have roughly equal intensities, it is concluded that Bi is the second layer down in both types of region. Thus, Te and I are thought to form the surface terminations. These candidates for the two terminations are consistent with those expected from the bulk crystal structure, where cleavage along the van der Waals bonded planes would leave Te or I as the terminating layer, and Bi buried beneath.

### DFT calculations and tunnelling spectroscopy

With these expectations as to the surface terminations present, we explicitly connect the chemical identities of the terminating layers to the spatial distribution of the terminations observed using STM. Tunnelling spectra taken from each termination were compared with the PDOS of the uppermost layer for each case. The choices for the uppermost layer in each calculation model were informed by the XPS result described above and by the expected terminations based on the bulk crystal structure and bonding.

**Figure 3:**
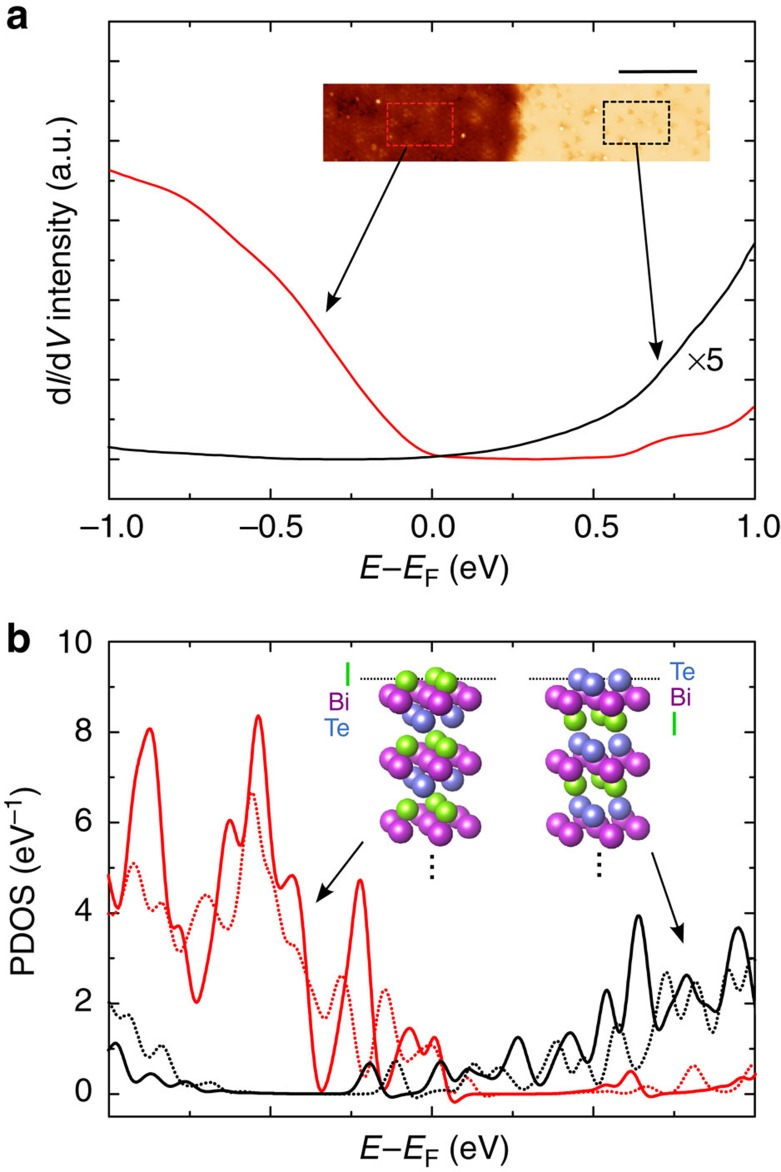
A comparison of measured tunnelling spectra with calculated PDOS for expected BiTeI terminations. (**a**) Spatially averaged STS curves taken in the rectangular areas bounded by dashed lines in the STM topography map (inset, with a scale bar of 20 nm). The black curve has been enhanced by a factor of five for ease of comparison. The discrepancy in curve intensities is thought to arise from the density-of-states contrast between the terminations resulting in different tip-to-sample distances determined by the tunnelling feedback set-point (*V*_bias_=0.7 V, *I*_set_=0.3 nA). (**b**) PDOS calculated for the uppermost atomic layer for the relaxed (solid curves) and unrelaxed (dashed curves) surfaces terminated by Te (black curves) and I (red curves). The atomic structure models for the two terminations are illustrated in the inset.

The PDOS results for the Te and I terminations each exhibit a pronounced shifting of the band gap from the bulk position (see Supplementary Fig. 2), with a negative energy shift for the Te termination and a positive shift for the I termination. Comparing the direction of shift obtained by STS measurement (Fig. 3a) and by calculated PDOS (Fig. 3b), we conclude that the raised surface seen in STM topography maps is the Te terminated one and that the recessed surface is I terminated.

At this point, it is worth noting that since the PDOS has been calculated for each atomic layer including the uppermost Bi layers in both types of surface (see Methods section for details), the energy shift obtained using DFT calculations can be checked against the shift observed in the XPS spectra. As an energy marker in the PDOS which is common to Bi in both surfaces, we choose the minimum level found in the conduction band, which is known to be dominated by the 6p states, and observe a shift of around 0.8 eV between states in the two surfaces (see Supplementary Fig. 3), in line with the shift of the Bi 5d states shown in the XPS spectrum of Fig. 2c.

### Spatially resolved tunnelling spectroscopy

**Figure 4:**
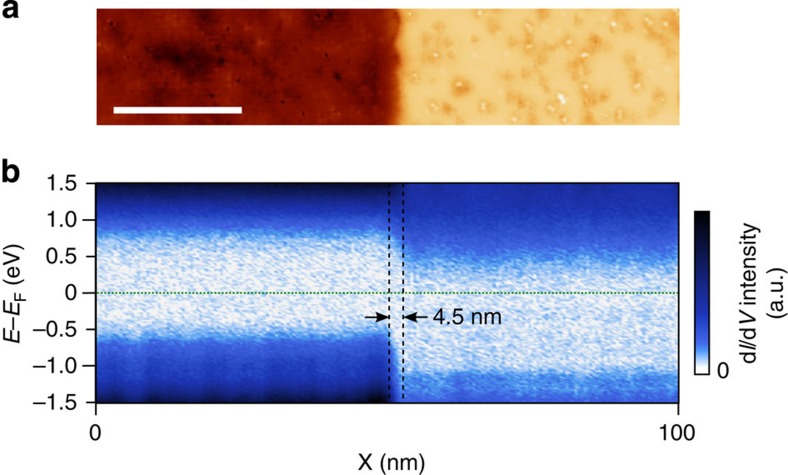
Spatially resolved tunnelling spectra. (**a**) STM topography showing the boundary between areas of I (left) and Te (right) terminated surface (*V*_bias_=0.7 V, *I*_set_=0.3 nA). Scale bar, 20 nm. (**b**) Spatially resolved STS measurements (averaged over the short axis of the topography image). Though the starting set-point chosen here (*V*_bias_=1.5 V, *I*_set_=0.3 nA) yields curves less representative of the DOS than those shown in Fig. 3 (with a set-point at *V*_bias_=0.7 V, *I*_set_=0.3 nA), this set-point is chosen to attain approximate parity in signal intensity between curves taken from each termination, with an emphasis on resolving the lateral behaviour at the junction. Vertical dashed lines enclose the transition region between the characteristic electronic structures of the two terminations.

To directly visualize the nano-scale evolution of the local electronic structure across the boundary between areas of different terminations, spatially resolved STS measurements were performed, as shown in Fig. 4. Tunnelling spectra were acquired along a path crossing the boundary and compared with the corresponding topography map (Fig. 4a). The shifting of the semiconductor band-gap is clearly observed, with a transition corresponding to the topographic step between the two regions.

## Discussion

The spatially resolved tunnelling spectra reveal p-n junction-like electronic structure at the lateral boundary between surfaces of Te and I terminations. As confirmed by PDOS results, this boundary represents a type of lateral junction where the relative position of the Fermi level is shifted with respect to the band edges, due principally to the surface polarization, rather than by distribution of doping concentrations. The junction is sharp, with a transition from p-like to n-like electronic structure over a distance of ~4.5 nm.

Far from the boundary, the positive surface potential at the Te termination (negative at the I termination) is screened by electrons (holes), forming an accumulation (depletion) layer. The vertical extent of the depletion and accumulation layers are each seen in DFT calculations (Supplementary Fig. 2) to be 2~3 nm. At the boundary, we expect a mutual screening of each side’s surface potential by re-arrangement of each side’s carriers (a transfer of electrons from the Te terminated region to the I terminated one), analogous to the formation of the space charge region of a p-n junction. Because of the ionic nature of the interlayer bonding on each side, there is unlikely to be significant intermixing at the boundary, and the stacking fault is expected to be atomically sharp. In this case, the observed transition reflects the scale of the space charge region between the p-like and n-like terminations. Interestingly, a scale of 4.5 nm corresponds well with the summed length scales of the depletion and accumulation layers for the two surfaces (each 2~3 nm). This indicates comparable screening lengths in the **ab** plane and along the **c** axis, which is peculiar considering the expected anisotropy of the free carrier effective mass^19^.

Furthermore, as the valence band in the p-like region and conduction band in the n-like region are both shown to exhibit Rashba spin splitting (see Supplementary Figs 4 and 5), the observed nanostructure may represent a Rashba p-n junction, as previously speculated upon by Crepaldi *et al*.^16^, and could open the possibility for related device concepts.

Control of the spatial distribution of the terminations is key to application in devices. Previously, the spatial distribution of the I and Te terminated surfaces of BiTeI have been attributed to cleavage through stacking faults, which reverse the stacking sequence along the **c** axis, and hence determine which layer is the uppermost after cleavage along a van der Waals bonded plane. Uniform terminations thought to arise in this way have been observed on a scale of around 100 μm by Crepaldi *et al*.^16^, using effective work function contrast in photoelectron emission microscopy. In this work, the terminations were instead observed to be mixed on the nano-scale. Their appearance on cleaved BiTeI surfaces was seen to be insensitive to variation in the cleavage temperature, ranging from 10 K to 300 K, and also to room temperature annealing after low temperature cleavage. A likely origin of this nano-scale reversal of stacking sequence is the presence of two mixed crystallites, fixed into the crystal during growth, whose lattices differ only by an inversion along the **c** axis. This model is pictured in Supplementary Fig. 6 along with the possible vertical alignment between the layers on either side of the stacking faults between domains, which can be elucidated from STM line profiles across the boundary. It is noted that the origin of the apparent step height across the boundary is found to be mostly topographic, rather than spectroscopic, as demonstrated by voltage-dependent morphology measurements presented in Supplementary Fig. 7.

The inversions between domains of each stacking order may arise due to the similar atomic number and electronegativity of Te and I, leading to the ease of a reversal of stacking during growth. The **ab** lattice orientation was seen to be uniform regardless of termination in both LEED and high-resolution STM measurements. The near 1:1 ratio of the two terminations, as found by analysing a large collection of STM conductance maps, is consistent with this picture, as the growth environment of the crystal does not force a breaking of spatial inversion symmetry and a resulting preference for one stacking sequence over the other.

In conclusion, we present the spectroscopic observation and microscopic mapping of termination dependent band bending at the surface of the Rashba semiconductor BiTeI. The two possible terminations, through their surface polarizations, induce energy shifts of electronic bands in the vicinity of the surface, with opposite shifts for positively charged Te terminated and negatively charged I terminated surfaces. This work provides microscopic identification of each of the two possible terminations of BiTeI, enabling detailed investigations into the surface defect chemistry for each termination. Furthermore, we have directly observed a Rashba p-n junction-like nanostructure, by monitoring the evolution of electronic structure across the lateral boundary between the two terminations utilizing spatially resolved tunnelling spectroscopy. This demonstrates a possible avenue for design of electronic properties in nano-scale Rashba spin-split systems by selection of polar surface terminations and control of their spatial distributions.

## Methods

### Crystal growth

BiTeI single crystals were grown by the CVT and melt growth methods and characterized by X-ray diffraction. To avoid potential explosion due to the formation of high pressure iodine vapour, the iodine must be premixed homogeneously with an additional room temperature agglomeration procedure. Melt growth involved the direct melting and solidification of a stoichiometric mixture of purified elements Bi, Te and I, which was sealed in an evacuated quartz ampoule, heated to 600 °C to soak for 24 h and slowly cooled to 400 °C at a rate of 1.5 °C h^−1^. The CVT growth was done through iodine vapour transport from the precursor with a molar ratio of Bi:Te:I=1:1:1.1, which was sealed in an evacuated quartz ampoule of 30 cm length and 1.8 cm inner diameter. Single crystals 0.5 × 0.5 × 0.1 cm^3^ in size, with large mirror like (001) surfaces, were obtained through vapour transport from the hot zone at 550–525 °C to the cold zone at 500 °C within 10 cm.

### Crystal cleavage

Cleavage of BiTeI crystals was performed in a preparation chamber with a base pressure lower than 5 × 10^−11^ mbar. Crystals were cleaved at room temperature or on a cryostat that could be cooled using LN_2_ or LHe, allowing cleavage temperatures of 70 K or 8 K, respectively. In the time between cleavage on the cryostat and measurement by low temperature STM, samples were transferred at room temperature. To preclude the possibility that any features observed were only the result of a thermally activated reconstruction on annealing to room temperature^20^, additional measurements were performed on crystals cleaved *in situ* on the the STM measurement stage, guaranteeing that the sample temperature remained below 10 K, from cleavage until STM measurement at 4.5 K. After cleavage, LEED patterns displaying a clear sixfold symmetry were obtainable over the entire sample surface, indicating a single homogeneous lattice orientation in the basal plane.

### STM measurements

All STM measurements were performed at a temperature of 4.5 K in an Omicron Low-Temperature STM using a tungsten tip. d*I*/d*V* intensity was mapped simultaneously with constant current topography images, and d*I*/d*V*(*V*) curves acquired simultaneously with *I*(*V*) curves, both using the lock-in technique with a bias modulation of 40 mV at a frequency of 5.9 kHz.

### XPS measurements

XPS measurements were performed using a scanning photoemission microscope (SPEM) at beamline 09A1 of the Taiwan Light Source^21^,^22^,^23^. The photon energy was 380 eV, and the beam was focused using a Fresnel zone plate to a width of ~200 nm. Spectromicroscopy images taken at the C 1 s feature were used to search for areas of the sample surface free of contamination by ambient conditions, which may be caused by pre-existing fractures in the crystal, or by premature *ex vacuo* cleavage. Spectra were then obtained from well-cleaved surfaces by zooming in to regions with a near-zero C 1 s signal. The width of the beam attainable with SPEM was not sufficiently small to spatially resolve areas of uniform termination. Instead, the beam was incident on both terminations, resulting in an XPS signal with mixed contributions from each, though in general the two terminations would not be evenly represented under the beam. The XPS measurement was performed in five different positions on the sample. Assuming a beam diameter of 200 nm gives a total sampling area of 1.6 × 10^5^ nm^2^. As mentioned in the description of STM results, the ratio of coverage of the terminations has been found to approach unity for surface areas of this size. Hence it is expected that the averaged XPS signal over the five positions is representative of an even mixture of both terminations. A fitting curve, synthesized using the Voigt profile in the UNFIT simulation package, was used to separate the two terminations’ contributions as seen in Fig. 2c.

### DFT calculations

First principle calculations of the surface PDOS for the two terminations were performed in accordance with DFT using the QUANTUM-ESPRESSO package^24^, with the Perdew-Burke-Ernzerhof exchange correlation functional. Relativistic effects including spin-orbit coupling were accounted for. Slab models of thirteen BiTeI trilayers were constructed, and the band structures and PDOS of each layer of the slab were calculated. Finally, the uppermost two trilayers of each slab were allowed to relax, taking into account the van der Waals interaction, and the PDOS calculated again. After relaxation, the topmost interplanar spacing between first and second layers of both Te and I corresponded closely to the value obtained experimentally at the single-unit-cell step edge in STM topography maps, though the relevant features of the final calculated PDOS were found to be largely insensitive to such structural relaxation. Exclusion of the van der Waals interaction from the calculation resulted in a further expansion of the spacing between trilayers near the surface. For the I terminated structure, the spacing between the Te layer of the uppermost trilayer and the I layer of the trilayer beneath increased from *d*_Te−I_=3.33 Å with the van der Waals interaction to *d*_Te−I_=3.57 Å without it, representing an expansion of 7.2%. This resulted in a total spacing between the first and second I layers of 7.46 Å, which is significantly larger than the experimentally relevant value as shown in Fig. 1e.

## Author contributions

C.J.B and M.-T.L. designed and prepared the experiment. C.J.B. performed STM and STS measurements with assistance from H.-H.Y., and support in instrument maintenance from C.-I.L. XPS measurements using SPEM were performed by C.J.B., J.-Y.H. and K.-H.O.Y. at the TLS, National Synchrotron Radiation Research Center, with assistance from SPEM instrument scientist H.-W.S. and endstation spokesperson C.-H.C. XPS data analysis was performed by J.-Y.H. Crystal growth and characterization were performed by R.S. under the supervision of F.-C.C. DFT calculations were performed by S.-H.H., with additional surface band structure calculated by H.-Y.L., both under the supervision of C.-C.K. The manuscript was prepared by C.J.B. and M.-T.L., with input from G.-J.S. and F.-C.C. The project was conceived and led by M.-T.L.

## Additional information

**How to cite this article:** Butler, C. J. *et al*. Mapping polarization induced surface band bending on the Rashba semiconductor BiTeI. *Nat. Commun.* 5:4066 doi: 10.1038/ncomms5066 (2014).

## Supplementary Material

Supplementary Information Supplementary Figures 1-7 and Supplementary Reference
