# Supplementary material for: Mapping polarization induced surface band bending on the Rashba semiconductor BiTeI
Source: Nat Commun. 2014 Jun 5;5:4066. doi: 10.1038/ncomms5066 (PMC4059917; doi:10.1038/ncomms5066)
Supplement: Supplementary Information — Supplementary Figures 1-7 and Supplementary Reference [file ncomms5066-s1.pdf]

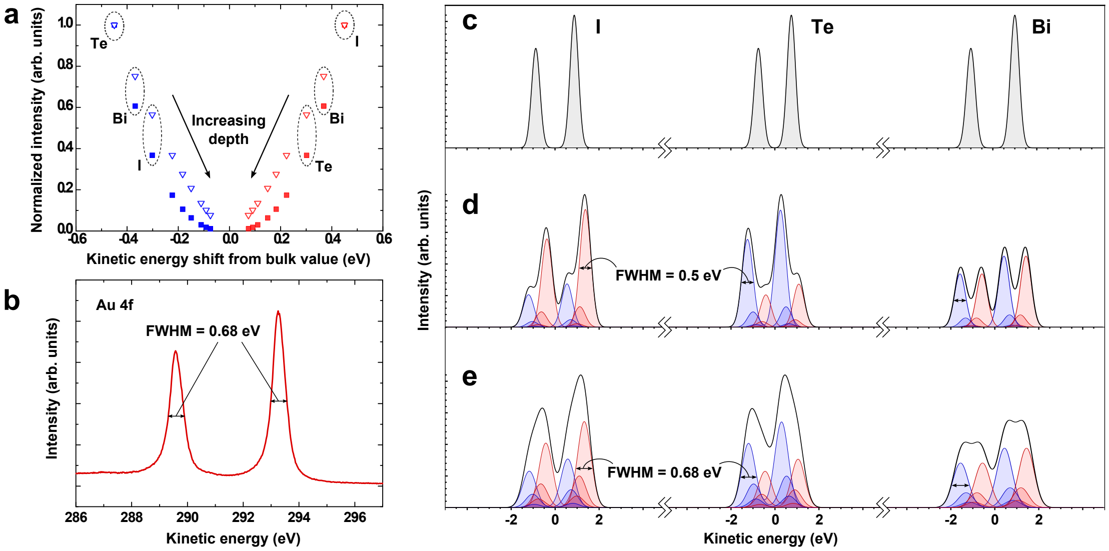

Supplementary Fig. 1. The effects of photoelectron inelastic mean free path (IMFP) and spectral resolution in different XPS measurements. (**a**) The variation of signal intensity with layer depth, controlled by the IMFP, and surface polarization induced energy shift plotted for each layer in the Te-termination (blue) and I-termination (red). Solid square points show the signal attenuation for a photon energy of 120 eV as used by Crepaldi *et al.*,[1] while open triangles show attenuation for a photon energy of 380 eV as used in this work. A suitable decay constant is chosen so that the energy shifts approach zero below 2 nm (about 3 unit cells). (**b**) The Au 4f spectrum showing the instrument spectral resolution of the SPEM at the time of this work. (**c**) Functions standing in for the idealized raw Bi 5d, Te 4d and I 4d doublets used in the model. (**d**) Modeled spectra considering photon energy of 120 eV and approximate spectral resolution of 0.5 eV, as estimated from the work by Crepaldi *et al.*[1] Curves shaded in red show those contributions originating from I-terminated regions, and curves shaded in blue show those from Te-terminated regions. (**e**) Modeled spectra in the case of the photon energy of 380 eV and spectral resolution of 0.68 eV, as attainable using the SPEM instrument.

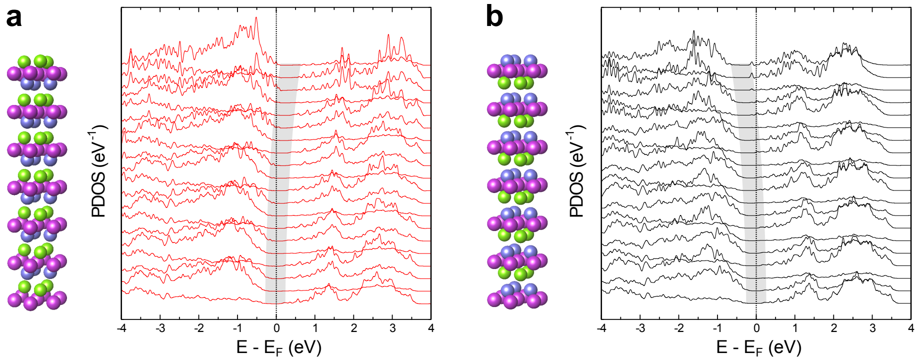

Supplementary Fig. 2. Projected density of states (PDOS) for each layer in the (**a**) I terminated and (**b**) Te terminated surfaces. The uppermost curve corresponds to the termination layer, and the lowest curves represent the bulk. The grey stripe highlights the bending of the band-gap near the surface.

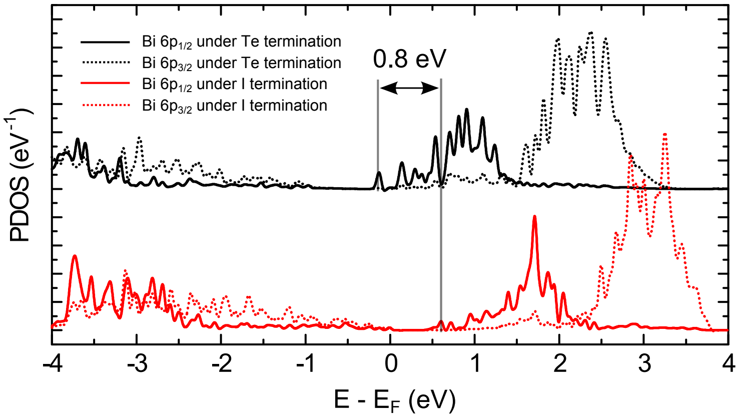

Supplementary Fig. 3. PDOS for the Bi 6p levels in the Bi layer closest to the surface (buried by the I or Te termination layer), showing a clear energy shift. Taking the conduction band minimum as a common marker, a shift of 0.8 eV is obtained, consistent with the shift seen for Bi 5d levels in photoemission spectra.

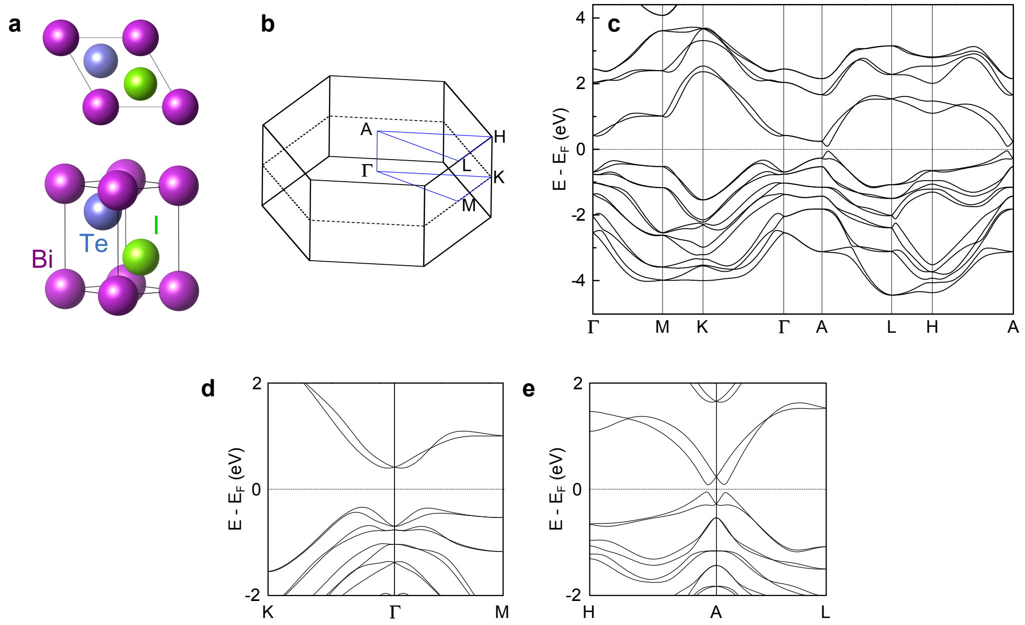

Supplementary Fig. 4. Bulk band structure. (**a**) The BiTeI unit cell and (**b**) first Brillouin Zone. (**c**) Calculated band structure for bulk BiTeI, after relaxation of the bulk structure with van der Waals interaction taken into account. (Inclusion or exclusion of the van der Waals interaction in the calculation yielded no significant differences in the resulting bulk band structure.) (**d**) and (**e**) Zoom-in on the band structure around the Γ and A points respectively, showing giant Rashba spin splitting.

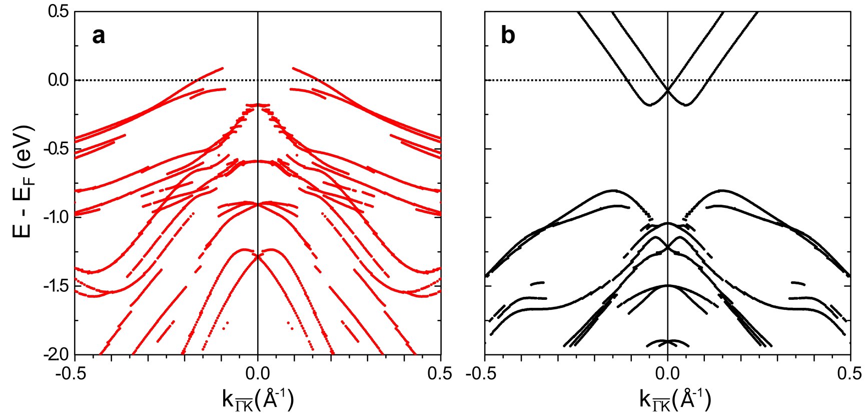

Supplementary Fig. 5. Calculated band structures for the uppermost atomic layer at (**a**) the I terminated and (**b**) the Te terminated surfaces. Only points for which the projection amplitude summed over all relevant orbitals exceeds 0.1 are included.

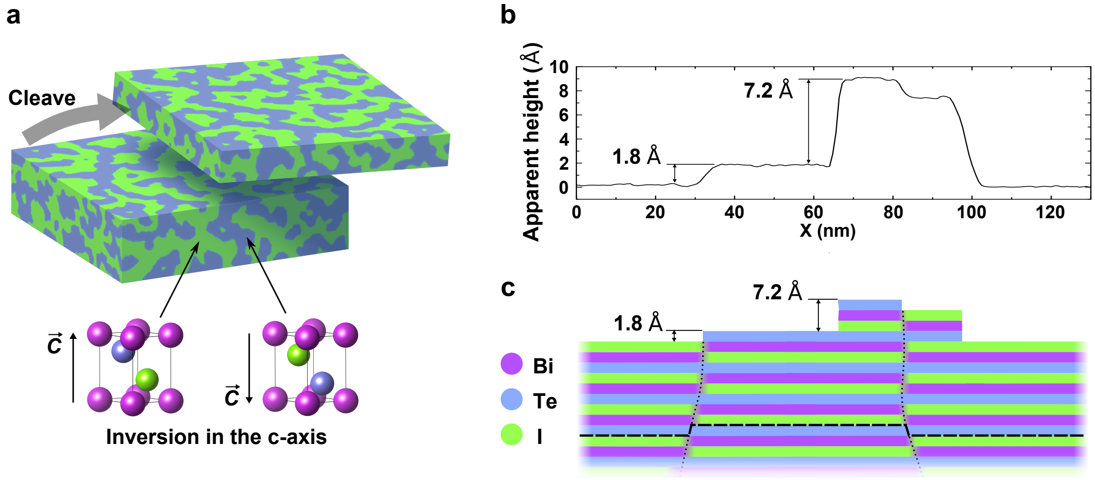

Supplementary Fig. 6. A proposed scheme for the occurrence of domains of inverse stacking order in crystals of BiTeI used in our work. (**a**) Stacking faults arranged on irregular three-dimensional surfaces throughout the crystal bulk separate domains of inverse stacking order. (**b**) Topographic line profile shown in Fig. 1, showing two distinct topographic step heights and (**c**) a possible model for the corresponding arrangement of the stacked layers on either side of stacking faults which extend into the bulk. The dotted lines indicate the position of the stacking faults. The dashed line indicates another possible path for cleavage of the crystal between along the van der Waals gap between the Te and I layers.

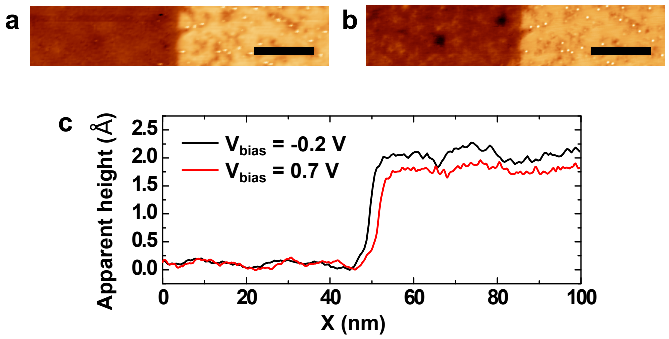

Supplementary Fig. 7. Voltage dependent morphology at the boundary between terminations. STM topography maps taken at (**a**) V_{bias} = -0.2 V, and (**b**) V_{bias} = 0.7 V, respectively. Both images are taken with a current set-point of 0.3 nA. Scale bars, 20 nm. (**c**) Averaged line profiles for the topography maps in (**a**) and (**b**).

Supplementary References

[1] Crepaldi, A. *et al.* Giant ambipolar Rashba effect in the semiconductor BiTeI. *Phys. Rev. Lett.* **109,** 096803 (2012).
